# Observational study over 8-year period evaluating microbiological characteristics and risk factor for isolation of multidrug-resistant organisms (MDRO) in patients with healthcare-associated infections (HAIs) hospitalized in a urology ward

**DOI:** 10.3205/id000073

**Published:** 2021-08-30

**Authors:** José Medina-Polo, Javier Gil-Moradillo, Alejandro González-Díaz, Pablo Abad-López, Rocío Santos-Pérez de la Blanca, Mario Hernández-Arroyo, Helena Peña-Vallejo, Julio Téigell-Tobar, Cristina Calzas-Montalvo, Prado Caro-González, Natalia Miranda-Utrera, Ángel Tejido-Sánchez

**Affiliations:** 1 Department of Urology, Health Research Institute i+12, Hospital Universitario 12 de Octubre, Madrid, Spain

**Keywords:** antibiotic resistance, healthcare-associated infection (HAI), multidrug-resistant organism (MDRO), urology department

## Abstract

**Objective:** To analyze, in a urology ward, the prevalence and characteristics of healthcare-associated infections (HAIs) due to multidrug-resistant organisms (MDRO).

**Methods:** We carried out an observational study from 2012 to 2019, evaluating MDRO among patients with HAIs, who were hospitalized in the urology ward. MDRO include *Pseudomonas *spp., resistant to at least three antibiotic groups, extended-spectrum beta-lactamase (ESBL) producing *Enterobacteriaceae* or those resistant to carbapenems, and *Enterococcus* spp. resistant to vancomycin.

**Results:** Among patients with HAIs, MDRO were isolated in 100 out of 438 (22.8%) positive cultures. Univariate and multivariate analyses reported that prior urinary tract infection (UTI) [OR 2.45; 95% CI 1.14–5.36; p=0.021] and immunosuppression [OR 2.13; 95% CI 1.11–4.10; p=0.023] were risk factors for MDRO. A high prevalence of MRDO was found in patients with a catheter in the upper urinary tract; 27.6% for double J stent, 29.6% in those with a nephrostomy tube, and 50% in those with a percutaneous internal/external nephroureteral (PCNU) stent. MDRO were isolated in 28.4% of cultures with *Enterobacteriaceae* (23.8% and 44.7% in those with *E. coli* and *Klebsiella* spp.); 7% of *Enterobacteriaceae* showed resistance to carbapenems (1.3% and 10% for *E. coli* and *Klebsiella* spp., respectively). Three out of 80 *Enterococcus* spp. were vancomycin-resistant. The rate of *Pseudomonas aeruginosa* resistant to at least three antibiotic groups was 36.3%.

**Conclusions:** The isolation of MDRO, in up to 25% of positive cultures in a urology ward, constitutes a challenge for the selection of antibiotics. MDRO are more common in immunosuppressed patients, those with previous UTIs, and those with a catheter in the upper urinary tract.

## Introduction

The prevalence of multidrug-resistant organisms (MDRO) has been increasing worldwide in recent years [1], [2]. Isolation from MDRO is of utmost importance in patients with healthcare-associated infections (HAIs) [3], [4], [5]. It is a public health warning that, far from being controlled, this seems to be one of the main challenges for medicine in the 21^st^ century. It is a problem that concerns all physicians and healthcare workers who manage patients with infections and use antibiotics. It is also necessary to bear in mind that when MDRO is isolated, there is limited availability of antibiotics to be prescribed [1], [6]. Thus, it seems of increasing importance for the detection of risk factors for HAIs and the isolation of MDRO to develop specific protocol and stewardship programs.

The prevalence of MDRO has been analyzed in some epidemiological studies [7]. However, data on the prevalence of MDRO in patients admitted to a urology service are limited. The main evaluation of MDRO in urology has been carried out by the GPIU study [4], [5], [8]. Urological patients have specific characteristics and specific risk factors, such as urinary catheters and surgery during hospitalization. Therefore, an adequate knowledge of risk factors, microbiological and resistance patterns for isolation of MDRO may allow select appropriate empiric antibiotic therapy and optimize outcomes [9]. Our purpose was to analyze the prevalence and characteristics of HAIs due to MDRO in patients admitted to a urology ward.

## Methods

We have carried out an observational study from January 2012 to December 2019, evaluating the prevalence and risk factors for isolation of MDRO in patients with HAIs admitted in a urology ward. The study collected information of all patients admitted in the urology ward from scheduled admission or the emergency department. Pediatric patients and those who underwent kidney transplantation are not included, since they are admitted to the pediatric urology and nephrology departments, respectively.

We collected demographic data of all the patients admitted to our department, such as age, gender, comorbidities such as arterial hypertension, diabetes mellitus, cardiovascular disease, liver disease, immunosuppression, and health condition using the American Society of Anesthesiologists’ (ASA) score. Regarding urological risk factors, we collected urinary lithiasis, previous urinary tract infection (UTI), a urinary catheter (urethral or in the upper urinary tract, during hospitalization or placed prior to admission). The prevalence of infections was assessed and defined clinically regardless of the results of the cultures. Healthcare-associated infections (HAIs) are defined according to the criteria of the Centers for Disease Control and Prevention as infection diagnosed 48 hours after admission or those with any of the following criteria: received parenteral treatment, specialized wound care, hemodialysis, or intravenous chemotherapy; those admitted to an acute hospitalization ward for at least two days in the last 90 days, or institutionalized in a residence or long-stay center.

Multidrug resistance was defined according to the ECDC and CDC definitions of multidrug resistance (MDRO), extensive drug resistance (XDR) and pan-drug resistance (PDR). MDRO was defined as acquired non-susceptibility to at least one agent in three or more antimicrobial categories, XDR was defined as non-susceptibility to at least one agent in all but two or fewer antimicrobial categories (i.e. bacterial isolates remain susceptible to only one or two categories) and PDR was defined as non-susceptibility to all agents in all antimicrobial categories [10]. We use the definition recommended by the ECDC and CDC which includes extended-spectrum beta-lactamase (ESBL) producing *Enterobacteriaceae* or those resistant to carbapenems, *Enterococcus* spp. resistant to vancomycin, and Methicillin-resistant *Staphylococcus aureus* (MRSA) and *Pseudomonas aeruginosa* resistant to more than three classes of antimicrobial agents. Pathogen identification, antimicrobial susceptibility testing and screening for ESBL and carbapenemase-producing bacteria were performed according to the cut-off point of resistance set by EUCAST (https://www.eucast.org). In brief, specimens were collected and processed following conventional microbiological procedures for correct management of clinical samples. Blood agar and MacConkey agar media were used. Identification and susceptibility tests were determined using the MicroScan^®^ panel. The initial screen test to indicate ESBL production was done using cefotaxime and clavulanic acid. The confirmatory test was carried out by using the double disk synergy method with the determination of the MIC testing ceftriaxone or ceftazidime and ceftriaxone/ceftazidime in combination with clavulanic acid. E-test (Epsilon test) was also performed in those cases in which results were not conclusive. Carbapenemase-producing bacteria was considered to be any enterobacteria in which the values of the minimum inhibitory concentrations of at least one carbapenem (imipenem, meropenem, doripenem, or ertapenem) were equal to or higher than the cut-off point of resistance. The type of carbapenemase-producing bacteria was also reported.

The type of HAIs was reported including healthcare-associated urinary tract infections (HAUTIs) or other types of HAIs such as surgical site infection, intraabdominal abscess, and vascular catheter associated infections. We also recorded if MDRO were isolated in blood, urine cultures, or samples from surgical site or abscess. The analysis included microbiological characteristics and risk factors for isolation of MDR microorganisms in urine or blood cultures. As the study was carried out in a period of 8 years, we also review the evolution over time.

Data were presented mainly as frequencies expressed as a percentage in categorical variables; for the quantitative variable, data were reported as mean and standard deviation. Risk factors for isolation of MDRO in patients with HAIs were assessed by univariate analysis using Chi-square tests or Fisher’s test for categorical variables and Student’s t-test for continuous variables. A multivariate analysis was then performed, including those variables with a p<0.1 in the univariate analysis, to find independent risk factors for the development of infection due to MDRO. Data are presented as frequencies, as well as odds ratio (OR) with 95% confidence interval (95% CI). The p<0.05 are considered statistically significant. The data has been collected and analyzed with the Statistical Package for Social Sciences version 23.0 (SPSS Inc., Chicago, IL., USA).

## Results

**Figure 1:**
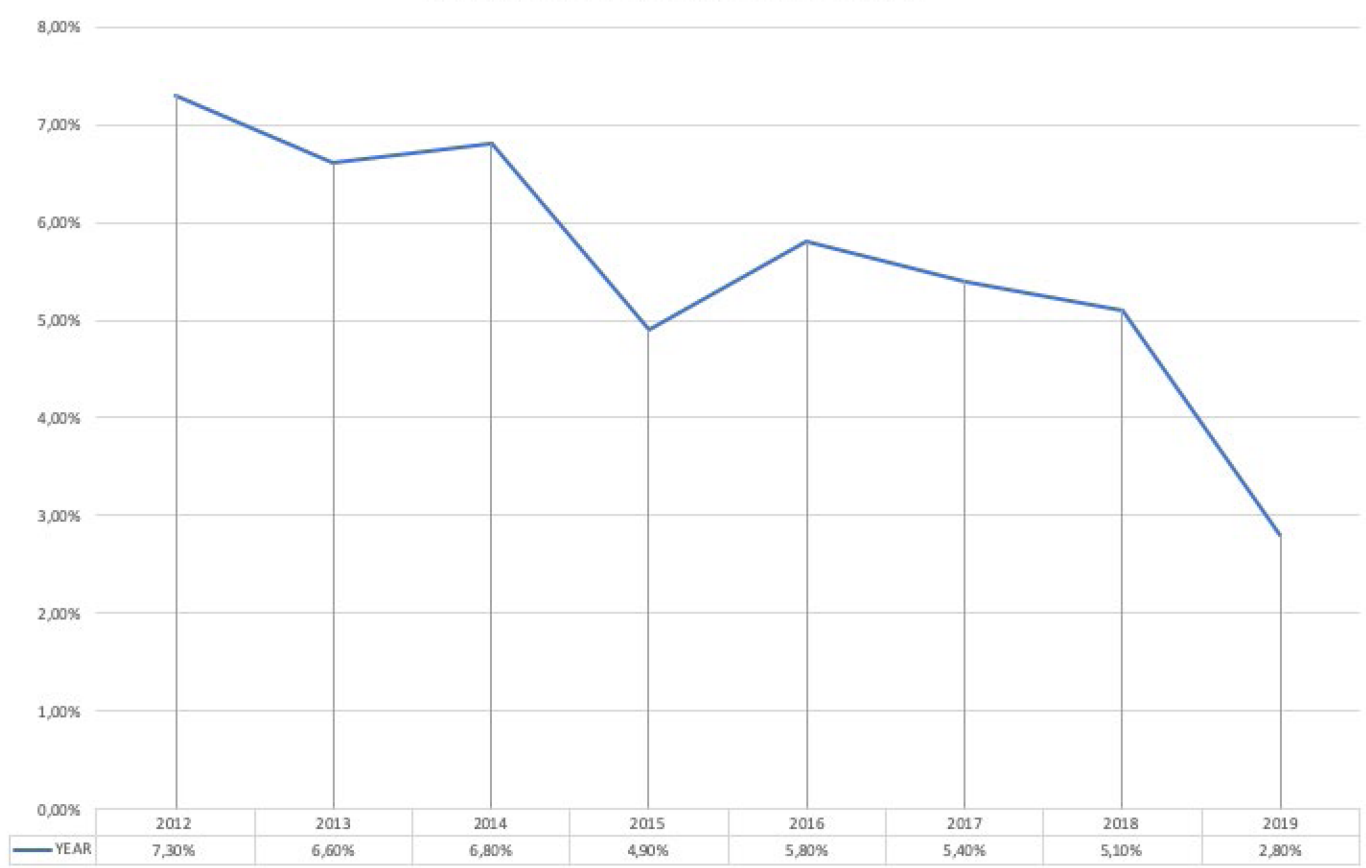
Evolution of the prevalence of HAIs in the urology ward from 2012 to 2019

**Figure 2:**
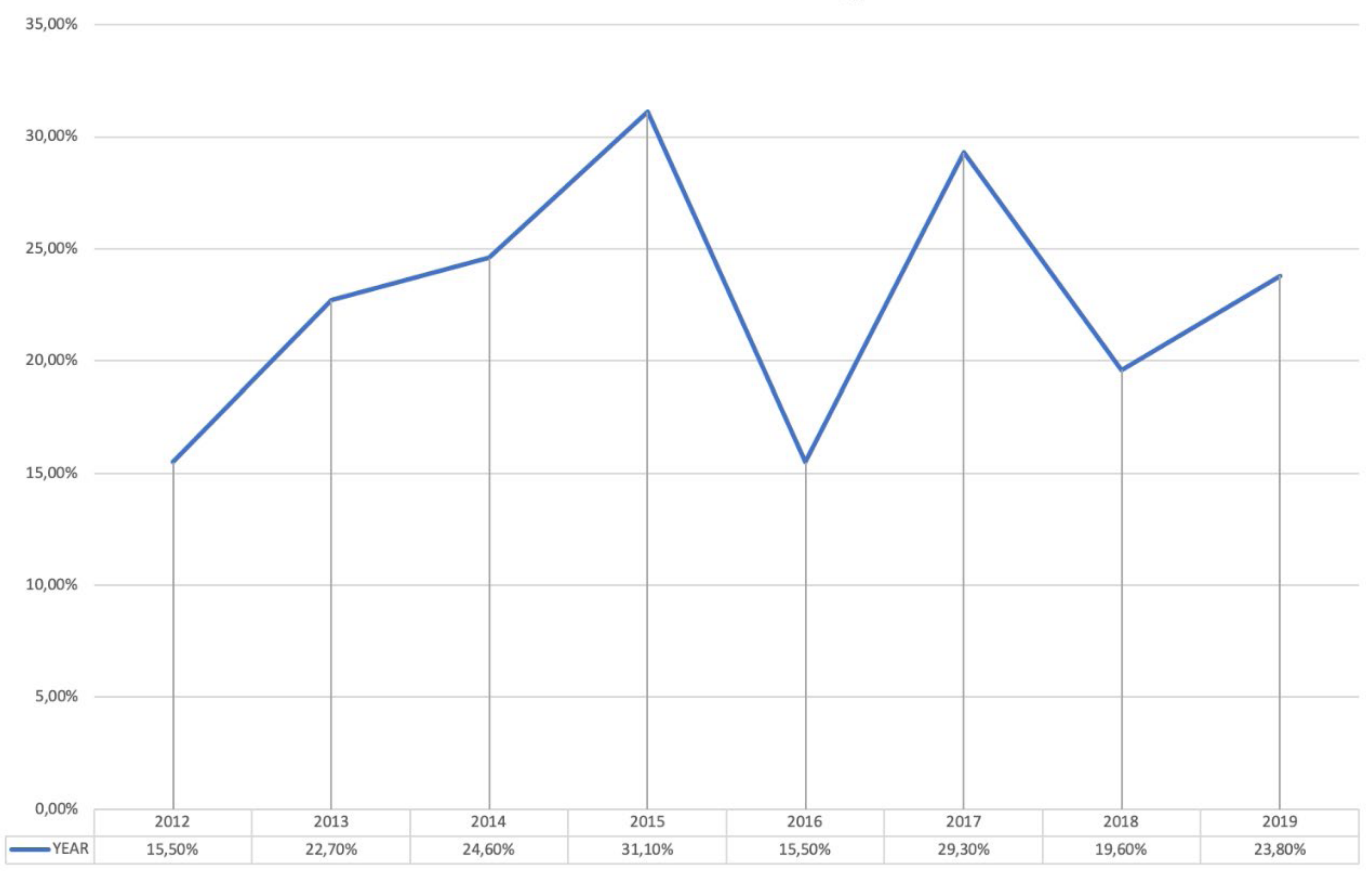
Evolution of the prevalence of multidrug-resistant microorganisms (MDRO) among patients with HAIs hospitalized in the urology ward from 2012 to 2019

The results indicated that 778 (5.5%) out of 14,224 patients admitted to the urology ward between 2012 and 2019 reported HAIs, with a prevalence that decreases from 7.3% in 2012 to 2.8% in 2019. The evolution of the prevalence of HAIs is shown in Figure 1 (Fig. 1). No microorganisms were isolated in 28.4% of patients. One microorganism was isolated in 42.9%. Several microorganisms were isolated in 19.7%. No culture was available or the result showed contamination in 9%. In patients with HAIs with positive cultures, the prevalence of MDRO microorganisms was 22.8%, with figures ranging from 15.5% in 2012 and 2016 to 31.1% in 2015. The evolution of the prevalence of MDRO is shown in Figure 2 (Fig. 2). HAUTIs were the most common types of HAIs (71.5%), followed by surgical site infections and intraabdominal abscesses, 17.5% and 11%, respectively. In patients with isolation of MDRO, the types of infections were HAUTIs in 81.7%, surgical site infections in 12.5%, and intraabdominal abscess in 5.8%. MDRO were mainly isolated from urine cultures (59%), followed by blood cultures in 22.3%, and samples from surgical site or intraabdominal abscess in 18.7%.

In patients with HAIs and a urinary catheter in the upper urinary, the prevalence of isolation of MDRO was 27.6% for those with a double J stent, 29.6% for those with a nephrostomy tube, and up to 50% in those with a percutaneous internal/external nephroureteral (PCNU) stent. The univariate analysis reported a higher prevalence of MDRO isolation in patients with arterial hypertension [OR 1.73; 95% CI 1.08–2.74; p=0.020], previous UTIs [OR 2.50; 95% CI 1.19–5.28; p=0.013] and immunosuppression [OR 2.25; 95% CI 1.19–4.24; p=0.010]. The results of the univariate analysis were summarized in Table 1 (Tab. 1). Apart from arterial hypertension, the previously mentioned factors were confirmed in a multivariable analysis. Urinary lithiasis was also found as a risk factor in the multivariate analysis (Table 2 (Tab. 2)).

MDRO were reported in 28.4% of patients where *Enterobacteriaceae* were isolated; 23.8% and 44.7% in those with *E. coli* (38 out of 160) and *Klebsiella* spp. (42 out of 94), respectively. No MDRO were reported in patients with isolation of *Citrobacter* spp. (0 out of 7) and *Proteus* spp. (0 out of 14). The percentages of MDRO in patients with *Enterobacter* spp., *Morganella* spp. and *Serratia* spp. isolation were 26.5% (9 out of 34), 21.4% (3 out of 14), and 50% (3 out of 6), respectively. Among cultures with the isolation of *Enterobacteriaceae*, 7% showed resistance to carbapenems. Two cases (1.3%) of *E. coli* resistant to carbapenems were reported. Among cultures with the isolation of *Klebsiella* spp., 10% were resistant to carbapenems. All carbapenemase-producing bacteria isolated from type OXA-48. Three out of 80 *Enterococcus* spp. were vancomycin-resistant. The rate of *Pseudomonas* *aeruginosa* resistant to at least three antibiotic groups was 36.3%. Table 3 (Tab. 3) reported the microbiological characteristics of the MDRO isolated. Cultures in which MDRO were isolated showed 75.8% and 64.8% susceptibility rates for carbapenems and amikacin, respectively. On the other hand, the cultures with the isolation of MDRO reported a resistance rate to fluoroquinolones of 84.2%. Finally, the empirical antibiotic treatment was not adequate in 28% of patients with MDRO in comparison with 13.2% of patients with HAIs due to non-MDRO.

## Discussion

One of the main consequences of antibiotics’ widespread use is the emergence of microorganisms with resistance to antibiotics. Nowadays, a primary concern for worldwide healthcare systems is the increasing prevalence of antibiotic resistance in HAIs and outpatient settings. Moreover, the emergence of MDRO is a worrisome point, as selecting an appropriate antibiotic treatment is a challenging task [11]. It is estimated that MDRO infections increase hospitalization costs by 10% to 30% [12], [13]. Our data showed that although continuous monitoring and prevention of infections has led to a decrease in the global prevalence of HAI, there is an increasing prevalence of the presence of MDRO. Specifically, 22.8% were positive cultures with the isolation of MDRO, and up to 7% of patients with isolation of *Enterobacteriaceae* were producers of carbapenemases. Previous data from our center confirms that carbapenemase-producing *Enterobacteriaceae* infections represented 3.6% of all healthcare-associated infections and 9.7% of those caused by enterobacteria [14]. The outbreak of carbapenemase-producing bacteria is one of the most worrisome points, as in this case, the correct selection of antibiotics is a challenging task [1], [3], [14]. The situation is especially serious in some Asian countries, such as India and China, with very high rates of carbapenemase-producing MDRO [15], [16].

Nevertheless, identifying risk factors associated with the isolation of MDRO plays a fundamental role and may add in the choice of empirical antimicrobial therapy, especially if we suspect that we are facing an MDRO with resistance to carbapenems [14], [17]. According to our results, we confirm that the presence of immunosuppression, prior UTIs and urinary catheter in the upper urinary tract are risk factors for presenting MDRO infections. Those factors are also related to a higher risk of HAIs [2], [4], [18]. However, we did not identify, among the possible risk factors analyzed, that the presence of diabetes mellitus or high anaesthetic risk (ASA score III–IV) were predisposing factors for the isolation of MDRO. Among urological patients, those with a catheter in the upper urinary tract (ureteral double J stent, nephrostomy tube, or percutaneous internal-external nephrostomy catheter) require special attention and show a high prevalence of infections due to MDRO [19], [20]. The risk of isolation of MDRO is especially high in those with a percutaneous internal-external nephrostomy catheter, up to 50% in our series.

One of the main issues when dealing with MDRO is the prescription of adequate empirical treatment [17], [21]. In our department, for those hospitalized in a urology ward with HAIs and risk factors for MDRO, the empirical treatment is based on carbapenems (the susceptibility rate was 63.8%) or aminoglycosides such as amikacin (the susceptibility rate was 66.3%). Aminoglycosides were the most common empirical treatment in penicillin allergic patients.

Hospital antibiotic stewardship programs and a continuous surveillance of infections are recommended in order to optimize the management of infections [22], [23], [24], [25]. Although the prevalence of MDRO is increasing globally, HAIs and MDRO infections must be evaluated locally and revised periodically in each center due to the variability of resistance with essential differences between continents, countries, and even centers in the same region [3], [26]. Therefore, based on clinical practice guidelines, each center has to collect its data on the prevalence of MDRO and design protocols for the prescription of empirical antibiotic therapy [11], [27], [28]. These measures are useful in order to optimize treatments and reduce complications, and will provide a better understanding of the development of resistance [13], [29]. Urologists must be aware that MDRO infections are common, and they are becoming increasingly aware. One of the main activities in urology to assess the characteristics of HAIs is the GPIU study [4], [5]. MDRO infections are a challenging task and may require management with the support of an infectious diseases specialist. However, as urologists manage the patients, they have to involve all diagnostic and therapeutic measures [30].

## Conclusions

MDRO are more common in immunosuppressed patients, as well as those with previous UTIs, urinary catheter before admission, and urinary catheters into the upper urinary tract. Therefore, the prevalence of MDRO in urology is a problem of great importance, since up to 25% of the isolates showed MDRO. The evaluation of the prevalence of MDRO is the first step towards developing strategies aimed at better control of HAIs, in addition to knowing the risk factors predisposed to presenting MDRO. This information should be used to determine which antibiotic therapy is the most appropriate. Due to the variability in the prevalence of MDRO infections, multicenter and transnational studies are necessary to develop joint strategies as well as consensus to counteract the progression of resistance to antibiotic therapy.

## Note

This article will also be published as a chapter of the Living Handbook “Urogenital Infections and Inflammations” [31].

## Acknowledgments

We acknowledge the effort and collaboration of all personnel of the department of urology for preventing infections. Moreover, special thanks to all the residents of our department who have actively collected and reviewed the data.

## Authors’ ORCIDs

- José Medina-Polo: 0000-0003-3626-8669
- Javier Gil-Moradillo: 0000-0002-8098-0218
- Alejandro González-Díaz: 0000-0002-3581-7200
- Pablo Abad-López: 0000-0001-7902-298X
- Rocío Santos-Pérez de la Blanca:
0000-0003-0063-6810
- Helena Peña-Vallejo: 0000-0001-6210-2318
- Julio Téigell-Tobar: 0000-0002-6873-8031
- Ángel Tejido-Sánchez: 0000-0001-8739-8370

## Competing interests

The authors declare that they have no competing interests.

## Figures and Tables

**Table 1:**
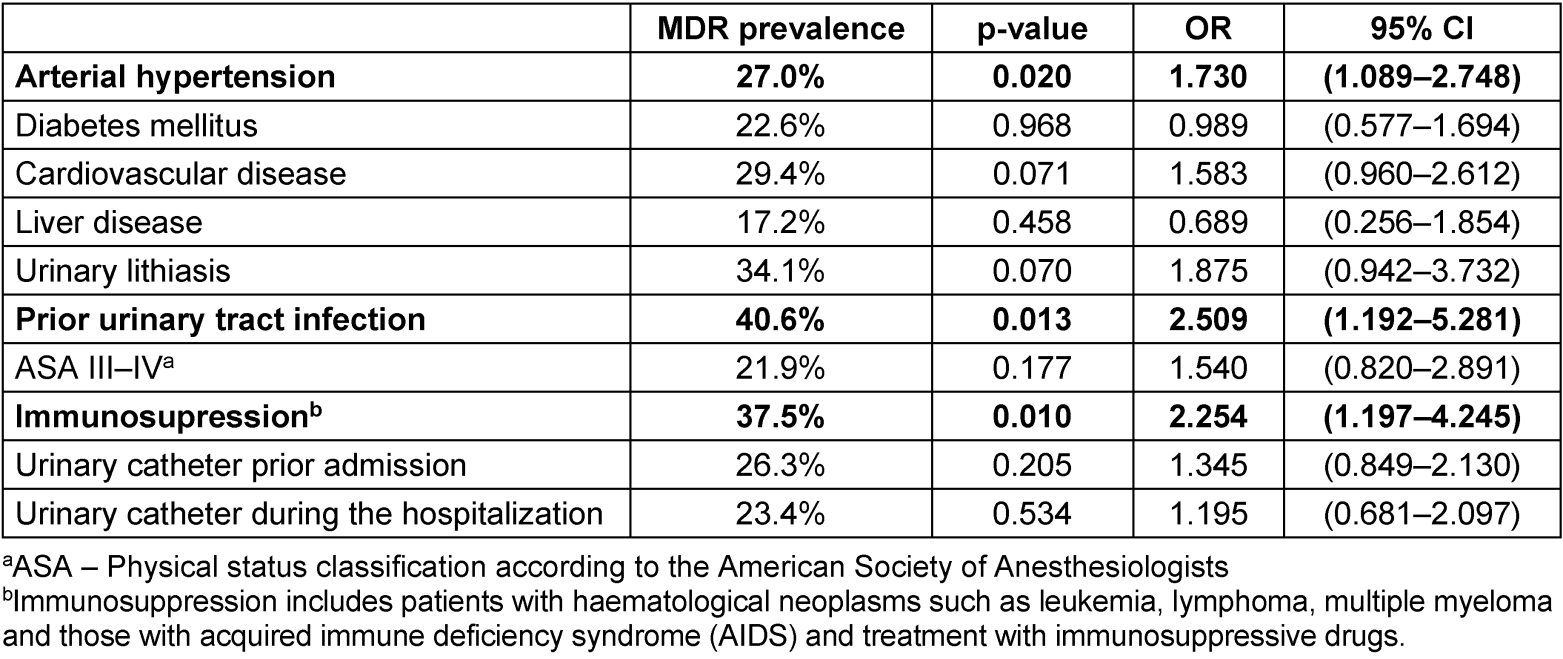
Univariate analysis of risk factors for isolation of multidrug-resistant organisms (MDRO) among patients with HAIs hospitalized in the urology ward

**Table 2:**
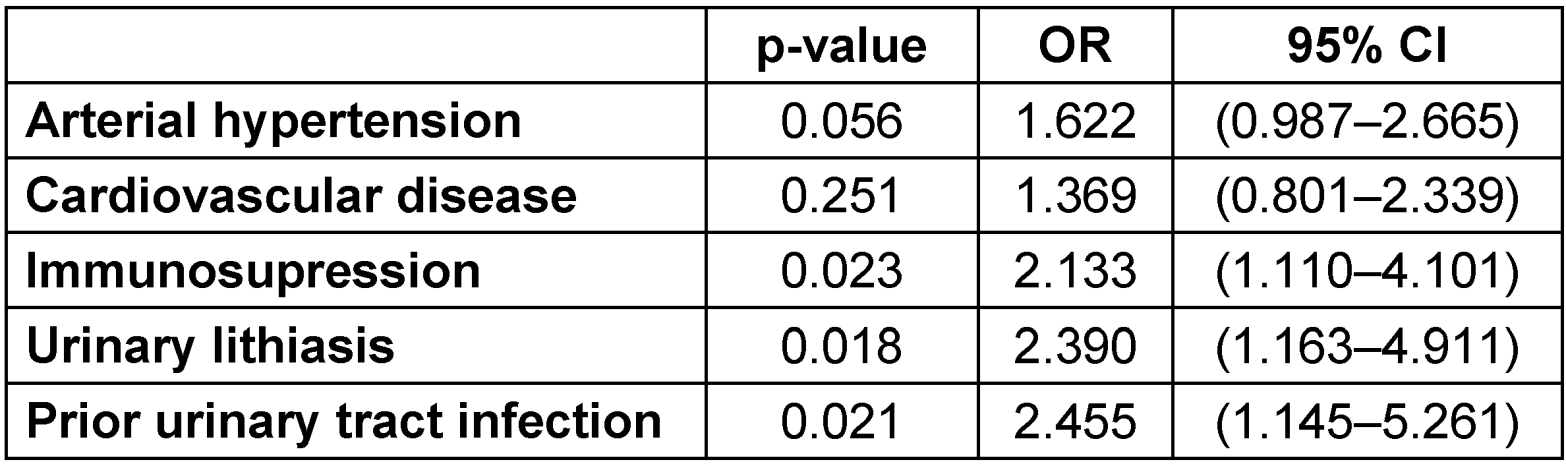
Multivariate analysis of risk factors for isolation of multdrug-resistant organisms (MDRO) among patients with HAI hospitalized in the urology ward

**Table 3:**
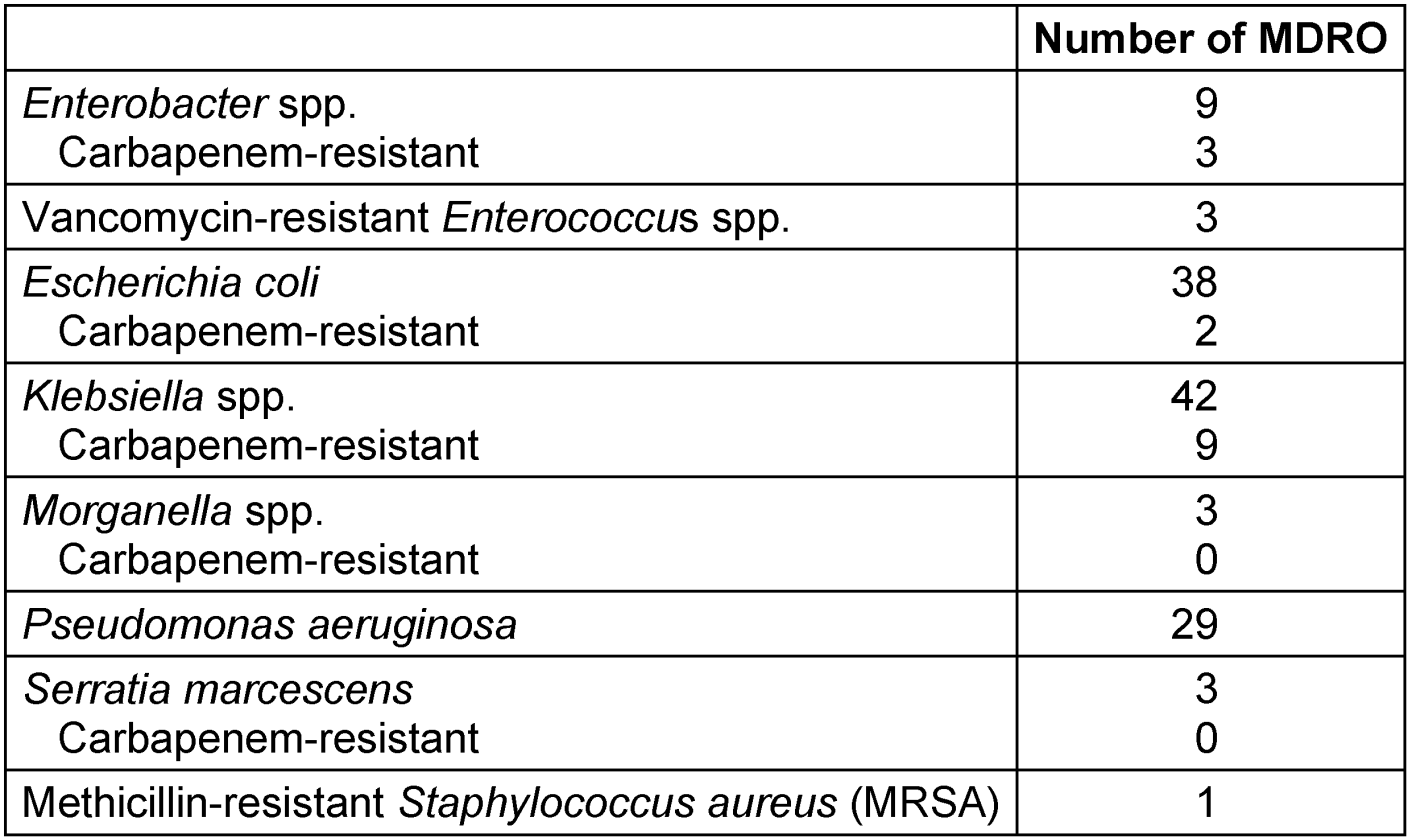
Multidrug-resistant organisms isolated among patients with HAIs hospitalized in the urology ward
